# Comprehensive Analysis of N6-Methylandenosine-Related Long Non-Coding RNAs Signature in Prognosis and Tumor Microenvironment of Bladder Cancer

**DOI:** 10.3389/fonc.2022.774307

**Published:** 2022-01-24

**Authors:** Kang Chen, Shaoming Zhu, Weimin Yu, Yuqi Xia, Ji Xing, Jie Geng, Fan Cheng

**Affiliations:** ^1^ Department of Urology, Renmin Hospital of Wuhan University, Wuhan, China; ^2^ Department of Urology, Suizhou Hospital, Hubei University of Medicine, Suizhou, China

**Keywords:** bladder cancer, N6-methyladenosine, prognostic signature, immune infiltration, long non-coding RNA

## Abstract

To investigate the role of N6-methyladenosine (m6A)- related long non-coding RNAs (lncRNAs) in bladder cancer (BC). 50 m6A-related lncRNAs were screened out and were correlated with prognosis from BC samples in The Cancer Genome Atlas (TCGA). The lncRNAs were subdivided into cluster 1 and cluster 2 with consensus cluster analysis, and it was found that lncRNAs in cluster 2 were associated with poor prognosis and increased PD-L1 expression. Gene set enrichment analysis (GSEA) revealed tumor-related pathways in cluster 2. Through least absolute shrinkage and selection operator (LASSO) Cox regression analysis, univariate and multivariate Cox regression, and ROC analyses, 14 prognostic lncRNAs were selected and used to construct the m6A-related lncRNA prognostic signature (m6A-LPS), furthermore, that m6A-LPS was as a valuable independent prognostic factor. Interestingly, the m6A-LPS risk score was positively correlated with the immune score, PD-L1 expression, and the infiltration of immune cell subtypes in BC. SNHG16, a member of the high-risk group based on m6A-LPS, was highly expressed in BC tissues and cell lines and interfered with siRNA resulted in suppressed proliferation, migration, and invasion *in vitro*. Our study illustrates the role of m6A-related lncRNAs in BC. The m6A-LPS may be an important regulatory target of the tumor microenvironment (TME) in BC.

## Introduction

Bladder cancer (BC) is the 10th most common cancer globally (1) and is characterized by high morbidity and mortality rates (2). Most BCs are urothelial carcinomas, of which approximately 75% are non-muscle-invasive and 25% are muscle-invasive or metastatic cancers (3). Non-muscle-invasive bladder cancer (NMIBC) has a high recurrence rate after resection and can develop into muscle-invasive bladder cancer (MIBC). MIBC has a poor prognosis and often recurs after the first resection (4). Although some advanced urinary assays could detect the early-stage bladder cancer leading to early treatment, the approach to predict the prognosis of bladder cancer and help clinical decision making is lacking and unsatisfied (5, 6). Despite the application of targeted therapy, immunotherapy, and antibody-drug conjugates for the treatment of advanced BC, the objective response rate (ORR) is still low (7). The immune checkpoint inhibitors (ICIs) treatments are currently applied during or flowing platinum-based chemotherapy, and also as the first-line therapy for the platinum ineligible metastatic bladder cancer (mBC). About 15-30% mBC is the response to ICIs treatment (8), indicating that the majority of patients do not benefit from ICIs. It is still a challenge to identify patients who will benefit from ICIs treatment and to improve the ORR. In addition, it is of great significance to predict and differentiate tumors of different subtypes based on tumor heterogeneity to individualize treatment approaches and improve the therapeutic effect while reducing the application of unnecessary treatment. However, the existing pathology-based diagnosis strategy does not reflect the intrinsic characteristics of the tumor. For example, even in the same pathological stage and grade of BC, the biological behavior of the tumor may be completely different (9). Although the molecular typing system can better reflect the intrinsic characteristics of BC than traditional pathological typing, it is not viable for clinical application because of its high cost and complexity. Therefore, the search for new economically viable and effective prediction methods is of great significance for improving the prognosis of BC and realizing individualized treatment.

N6-methyladenosine (m6A) is one of the most important modifications of RNA and mediates more than 60% of RNA methylation events (10). In general, m6A methylation plays an important role in the regulation of gene expression by modulating RNA splicing, translation efficiency, and mRNA stability (11). m6A modification is dynamic and reversible and consists of methyltransferase complexes (m6A “writers”), demethylases (m6A “erasers”), and m6A-binding proteins (m6A “readers”) (12, 13). In recent years, scientists have reported that m6A modification can affect tumor initiation and progression in various cancers, including BC (14). For example, METTL3 accelerates the maturation of pri-miR-221/222 in an m6A-dependent manner and promotes the proliferation of BC cells (14).

Long non-coding RNAs (lncRNAs) are non-protein-coding RNA molecules more than 200 nucleotides in length (15). In patient tumors, the abnormal expression of lncRNAs is a common biological phenomenon that is closely related to patient prognosis. For instance, the lncRNA metastasis-associated lung adenocarcinoma transcript 1 (MALAT1) was initially discovered in lung cancer, and the overexpression of MALAT1 is associated with a poor prognosis among patients with lung cancer (16). LncRNA LBCS inhibited the SOX2, stem cell factor, to suppress the self-renewal and enhance chemosensitivity in bladder cancer. In addition, lncRNA DANCR, BLACAT2, and LNMAT2 promote the lymphatic metastasis of bladder cancer (17–20). However, little research has been conducted on whether the expression of lncRNAs through m6A modifications affects the occurrence and development of BC. Therefore, understanding the role of lncRNAs modulated by m6A in BC helps identify biomarkers that can be used as meaningful therapeutic targets. However, the relationship between m6A-related lncRNAs and the response of BC to ICIs is still not fully understood, and the interaction between these lncRNAs and the tumor microenvironment (TME) needs to be further explored.

This study aimed to evaluate the correlation between m6A-related lncRNAs and the prognosis of BC. We identified 50 m6A-related lncRNAs related to the prognosis of BC and constructed a prognostic model for BC using bioinformatics methods in The Cancer Genome Atlas (TCGA) dataset. Furthermore, we investigated the possible roles of the selected m6A-related lncRNAs in the progression of BC by gene set enrichment analysis (GSEA) and single-sample gene set enrichment analysis (ssGSEA). In addition, we studied the relationships among these lncRNAs, programmed death-ligand 1 (PD-L1) expression, and the TME to determine whether the signature could be used to predict the response of BC patients to ICIs and immunotherapy.

## Materials and Methods

### Raw Data and m6A Gene Acquisition

We acquired BC transcriptome data and clinical data from TCGA (https://portal.gdc.cancer.gov/). The data for 434 BC samples, including 414 tumor samples and 19 normal samples, were downloaded. Nineteen normal samples were the paracancerous tissues of 19 of the 414 BC patients in the TCGA dataset. After excluding 6 duplicate samples and 2 samples without complete survival time and status, 406 of 414 tumor samples had complete clinical information and were included for further study. The clinical characteristics of these 406 patients with BC are shown in **Table 1**. According to previously published literature, we identified 23 m6A RNA methylation regulators based on TCGA BC dataset gene expression information. These 23 m6A regulators comprised 8 writers (METTL3, METTL14, METTL16, KIA1499, WTAP, RBM15, RBM15B, and ZC3H13), 2 erasers (ALKBH5 and FTO), and 13 readers (YTHDF1, YTHDF2, YTHDF3, YTHDC1, YTHDC2, HNRNPA2B1, HNRNPC, IGFBP1, IGFBP2, IGFBP3, FMR1, LRPPRC, and RBMX) (21).

**Table 1:** The clinical characteristics of BC patients in the TCGA datasets.

Characteristic	Type	n	Proportion (%)
Age	≤65	160	39.41%
	>65	246	60.59%
Gender	Female	107	26.35%
	Male	299	73.65%
Grade	High grade	383	94.33%
	Low grade	20	4.93%
	Unknown	3	0.74%
TNM stage (stage)	Stage I	2	0.49%
	Stage II	129	31.77%
	Stage III	140	34.49%
	Stage IV	133	32.76%
	Unknown	2	0.49%
T stage	T0	1	0.25%
	T1	3	0.74%
	T2	118	29.06%
	T3	193	47.54%
	T4	58	14.29%
	Unknown	33	8.13%
M stage	M0	195	48.03%
	M1	11	2.71%
	Unknown	200	49.26%
N stage	N0	236	58.13%
	N1	46	11.33%
	N2	75	18.47%
	N3	7	1.72%
	Unknown	42	10.34%

### Bioinformatic Analysis

Pearson correlation (|Pearson R| > 0.4 and *P* < 0.01) was implemented to identify lncRNAs correlated with m6A genes to identify m6A-related lncRNAs. Based on clinical survival data from TCGA datasets, we further identified m6A-related lncRNAs related to prognosis (p<0.01).

Patients with BC were divided into different subgroups by the ConsensusClusterPlus package (22) according to the expression of m6A-related lncRNAs related to prognosis. GSEA was performed, and hallmark gene sets were downloaded from the Molecular Signatures Database (MSigDB) (23); next, the enrichment results were selected based on the normalized enrichment score (NES) and a false discovery rate (FDR) value <0.05 to explain the survival differences between the different BC subtypes.

Next, the least absolute shrinkage and selection operator (LASSO) Cox regression was performed with the R package “glmnet” to construct the m6A-related lncRNA prognostic signature (m6A-LPS) (24). The risk score was calculated with the following equation: Risk score = $\sum_{i=1}^{n}Coefi*xi$ (*Coefi* represents the coefficients, and *xi* represents the fragments per kilobase of transcript per million mapped reads (FPKM) value of 50 m6A-related lncRNAs related to prognosis). According to this equation, we calculated the risk score for each BC patient. Then, taking the median risk score as the cutoff point, the patients were divided into a high-risk group and a low-risk group.

To estimate the prognostic capability of the risk score for 1-, 3-, and 5-year overall survival (OS), receiver operating characteristic (ROC) curves were carried out to assess the area under the curve (AUC) values (25). To determine the independent prognostic factors in BC, we analyzed the prognostic relationships between the risk score and gender, age, WHO grade, and stage based on univariate and multivariate Cox regression analyses. We then constructed a nomogram with integrated prognostic features to predict the 1-, 3-, and 5-year survival rates of patients with BC.

The immune score and stromal score of each sample were calculated in TCGA datasets using Estimation of Stromal and Immune cells in Malignant Tumor tissues using Expression data (ESTIMATE) algorithm via the “estimate” R package (26). For each sample, we quantified 22 types of infiltrating immune cells with cell type identification by estimating relative subsets of RNA transcripts (CIBERSORT) (27) and then selected samples with a CIBERSORT *P*-value <0.05 for subsequent analysis to compare differences in immune infiltration levels between the subgroups grouped by cluster subtype and risk score. In addition, we used ssGSEA to investigate the differences in immune cell infiltration and immune function between the high-risk and low-risk groups (28).

### Samples and Quantitative Real-Time Polymerase Chain Reaction

Thirteen nonneoplastic and neoplastic samples from patients who underwent surgical treatments were obtained from the Renmin Hospital of Wuhan University in 2019. To evaluate SNHG16 expression, we extracted total RNA from clinical BC samples using RNA TRIzol reagent (Invitrogen, Carlsbad, CA, USA). Reverse transcription was carried out using the iScript cDNA Synthesis Kit from Bio-Rad. qRT-PCR was conducted on a LightCycler^®^ 480 Real-Time PCR System (Roche, Germany). The relative lncRNA expression levels were calculated using the 2^-ΔΔCt method with GAPDH as an endogenous control. The primer sequences used were as follows: SNHG16 forward 5’- CCTCGTGCCAGTAACTCTGAAATC-3’ and reverse 5’- CTCAGTCACCAGAAACGAAACAC-3’; GAPDH forward 5’- GGAAGCTTGTCATCAATGGAAATC-3’ and reverse 5’- TGATGACCCTTTTGGCTCCC-3’.

### Cell Culture

The human BC cell lines 5637 and T24 and the immortalized human bladder epithelial cell line SV-HUC-1 were purchased from the Cell Bank of the Type Culture Collection of the Chinese Academy of Sciences, Shanghai Institute of Biochemistry and Cell Biology. 5637 and T24 cells were cultured in RPMI 1640, and SV-HUC-1 cells were cultured in F12K medium with 10% (Gibco, Grand Island, NY, USA) and penicillin-streptomycin (100 U/mL) at 37°C with 5% CO_2_.

### siRNA Transfection of Cell Line

5637 cells were transfected with small-interfering RNA (siRNA), and a negative control siRNA (Sangon Biotech, Shanghai, China) using 5637 was transfected with siRNA (Sangon Biotech, Shanghai, China) using Lipofectamine 2000 transfection reagent (Invitrogen). The siRNA sequences were 5’-UGGAAGAGCCUAAGAGGAATT-3’ (sense) and 5’-UUCCUCUUAGGGCUCUUCCATT-3’ (antisense). A negative control (NC) was transfected simultaneously with the siRNA. The NC sequences were 5’-UUCUCCGAACGUGUCACGUTT-3’ (sense) and 5’-ACGUGCCACGUUCGGAGAATT -3’ (antisense). The knockdown of SNHG16 expression following siRNA transfection was verified by qRT-PCR.

### CCK-8 Cell Viability, Scratch Wound Healing, and Transwell Invasion Assays

A total of 5637 cells were seeded into 96-well and 6-well plates. When the cells reached 30–50% confluence, they were transfected with either SNHG16 siRNA or the NC. At 0, 1, 2, 3, and 4 days after transfection, CCK-8 solution was added at a ratio of 1:9 to the 96-well plates, and optical density (OD) values at a wavelength of 450 nm were measured by a microplate reader. Transwell invasion assays were performed to detect the invasive ability of the cells. The upper layer of the transwell chamber was coated with Matrigel (BD Biosciences, USA); the cells were seeded into the upper chamber with medium without FBS, and the lower chamber was filled with complete culture medium. The cells were cultured in a humidified 5% CO2 incubator at 37°C for 24 hours. The invaded cells were fixed in 4% paraformaldehyde for 5 minutes and then stained with 0.3% crystal violet (Solarbio, China). The invading cells or migrating cells were counted under a light microscope.

### Statistical Analysis

Data were analyzed using R software version 4.0.2. Correlations between m6A genes and lncRNAs were analyzed by Pearson’s test, and a *P*-value <0.01 was considered significant. The log-rank test was used to compare Kaplan-Meier survival curves between various subgroups, including cluster subtypes and low- and high-risk subgroups. Group comparisons for continuous data were accomplished by Student’s t-test and one-way ANOVA. Categorical variables were compared using chi‐square tests. Univariate and multivariate Cox regression analyses were used to validate the independent prognostic factors for BC. A *P <*0.05 was considered significant.

## Results

### Acquisition of m6A-Related LncRNAs in BC

**Figure 1:**
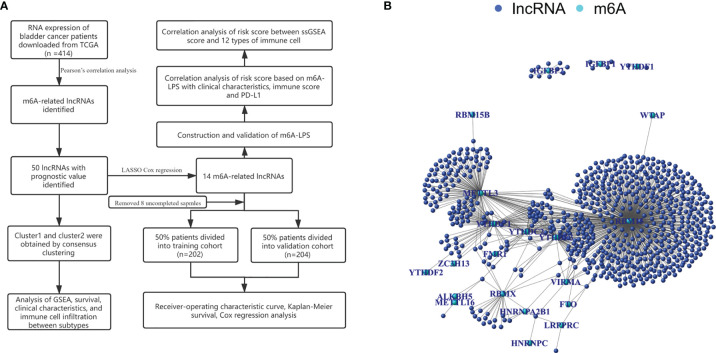
**(A)** Research flow chart. **(B)** The coexpression network of m6A-related genes and m6A-related lncRNAs.

**Figure 2:**
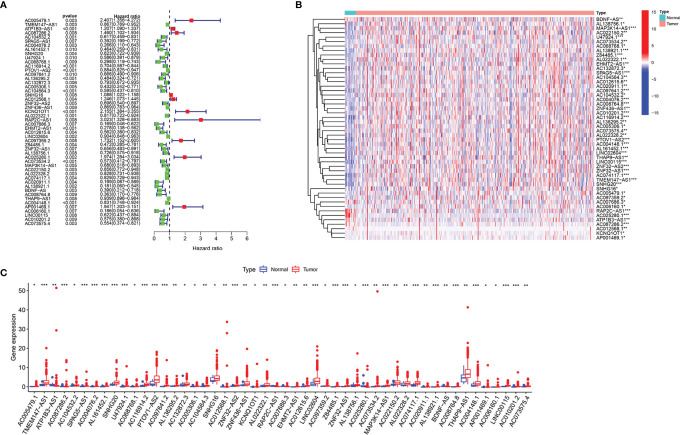
The differences in the expression of m6A-related lncRNAs in the TCGA datasets. **(A)** Forest plot of the hazard ratio for m6A-related lncRNAs. **(B, C)** Heatmap **(B)** and expression levels **(C)** of 50 m6A-related lncRNAs in tumor and adjacent normal tissues. *P < 0.05, **P < 0.01, and ***P < 0.001.

We identified 14,086 lncRNAs in the TCGA datasets by analyzing the annotated files downloaded from the GENCODE website. Then, 23 m6A gene expression matrices were extracted from the TCGA datasets. LncRNAs associated with one or more m6A genes (|Pearson R| > 0.4 and *P* < 0.01) were defined as m6A-related lncRNAs. Through Pearson’s correlation analysis, 414 lncRNAs were found to be significantly related to the m6A genes in the TCGA datasets. The specific process of this study is shown in **Figure 1A**. We obtained the coexpression network of m6A genes and m6A-related genes, as shown in **Figure 1B**. Next, according to the clinical survival information from the TCGA datasets, we screened 50 m6A-related lncRNAs with a significant prognostic value from the 411 m6A-related lncRNAs (*P* < 0.01). The risk ratio forest plot revealed that there were 40 low-risk and 10 high-risk m6A-related lncRNAs (**Figure 2A** and **Supplementary Table S1**). **Figures 2B, C** show the differences in the expression of m6A-related lncRNAs between 406 tumor tissues and 19 tumor-adjacent normal pairs from the TCGA dataset. Except for RAP2C-AS1, AC025280.1, ATP1B3-AS1, AC087286.2, AC012568.1, AC007686.3 and BDNF-AS, the other m6A-related lncRNAs were highly expressed in BC tissues (*P* < 0.05). These results suggested that these 50 m6A-related lncRNAs possess essential biological roles in BC development.

### Consensus Clustering Showed That m6A-Related LncRNAs Were Closely Associated With the Clinical Characteristics and Survival Rates of Patients With BC

**Figure 3:**
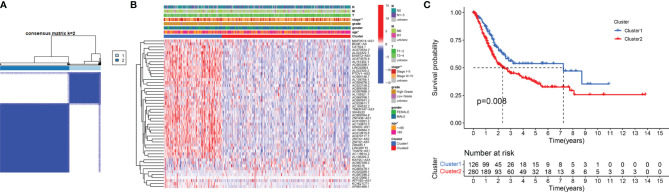
Differential clinicopathological features and survival rates of BC patients in cluster 1 and cluster 2. **(A)** Consensus clustering matrix for k = 2. **(B)** The heatmap of cluster 1 and cluster 2 along with clinicopathological features that included gender, age, grade, T stage, N stage, M stage, and stage. **(C)** Kaplan-Meier curves of OS. *P < 0.05 and **P < 0.01.

To verify the prognostic value of m6A-related lncRNAs, we conducted consensus clustering analysis on 406 BC samples using 50 m6A-related lncRNAs. Based on the similarity of the expression of m6A-related lncRNAs, we determined that K = 2 had the best clustering stability (K = 2 to 9). Then, we divided 406 BC samples into two subgroups, cluster 1 and cluster 2, for further analysis (**Figure 3A**). **Figure 3B** shows that most of the 50 m6A-related lncRNAs were highly expressed in cluster 2. The clinicopathological features between the two subtypes were then compared (**Figure 3B**). Cluster 2 mainly contained BC patients aged over 65 years (*P* < 0.05) and was preferentially associated with stage III–IV (*P* < 0.01). As shown in **Figure 3C**, the OS of cluster 2 was shorter than that of cluster 1 (*P* = 0.008), which indicated that the prognosis of the cluster 2 subgroup was poor compared with that of the cluster 1 subgroup. The similarity of the expression levels of 50 m6A-related lncRNAs in cluster 1 and cluster 2 samples showed that the expression level of these lncRNAs was closely related to the heterogeneity of the 406 BC patients in the TCGA dataset. Our results also confirmed the prognostic value of 50 m6A-related lncRNAs.

### GSEA of Cluster Subtypes and the Association Between PD-L1 and m6A-Related LncRNAs

**Figure 4:**
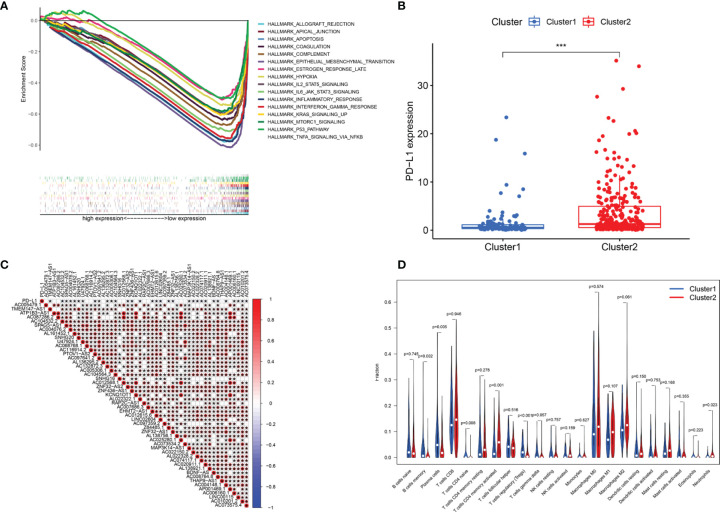
Association of PD-L1 with m6A-related lncRNAs and the landscape of immune cell infiltration in BC cluster 1 and cluster 2. **(A)** GSEA indicating that tumor hallmarks were enriched in cluster 2. **(B)** PD-L1 expression in BC cluster 1 and cluster 2. **(C)** The correlation of PD-L1 with m6A-related lncRNAs. **(D)** The infiltration levels of 22 immune cell types in BC cluster 1 and cluster 2. *P < 0.05; ***P < 0.001.

GSEA was performed to explore the potential regulatory mechanisms that led to the differences in survival rates between the cluster 1 and cluster 2 subgroups. The FDR of the enrichment analysis of cluster 1 was not <0.05. The enrichment analysis results of the cluster 2 subgroup revealed that allograft rejection, apical junctions, apoptosis, coagulation, complement, epithelial-mesenchymal transformation, delayed estrogen response, hypoxia, IL2-STAT5 signaling, IL6-JAK-STAT3 signaling, the inflammatory response, the interferon γ response, KRAS signaling, mTORC1 signaling, the P53 pathway and TNFA signaling through NFkB (FDR <0.05; **Figure 4A**) were enriched. Based on the correlation between the IL6/JAK/STAT3 signaling pathway and PD-L1 (29, 30), we analyzed the differential expression of PD-L1 between cluster 1 and cluster 2. Compared with that in cluster 1, PD-L1 expression in cluster 2 was significantly higher (p <0.001; **Figure 4B**). To explore the potential relationship between PD-L1 and m6A-related lncRNAs, we further studied the expression correlation between PD-L1 and m6A-related lncRNAs. The results showed that PD-L1 expression was correlated with that of most of the m6A-related lncRNAs, while the m6A-related lncRNAs showed a significant positive correlation with each other (**Figure 4C**). To analyze the potential relationship between m6A-related lncRNAs and the TME of BC, the level of immunocyte infiltration in cluster 1 and cluster 2 was evaluated, and we found that the infiltration level of naïve CD4 T cells, regulatory T cells (Tregs), memory B cells and plasma cells was relatively high in cluster 1, and that of neutrophils and activated memory CD4 T cells was relatively high in cluster 2 (**Figure 4D**). Hence, the IL6/JAK/STAT3 signaling pathway might be implicated in the distinct TME of cluster 1/2.

### Construction and Validation of the m6A-LPS

**Figure 5:**
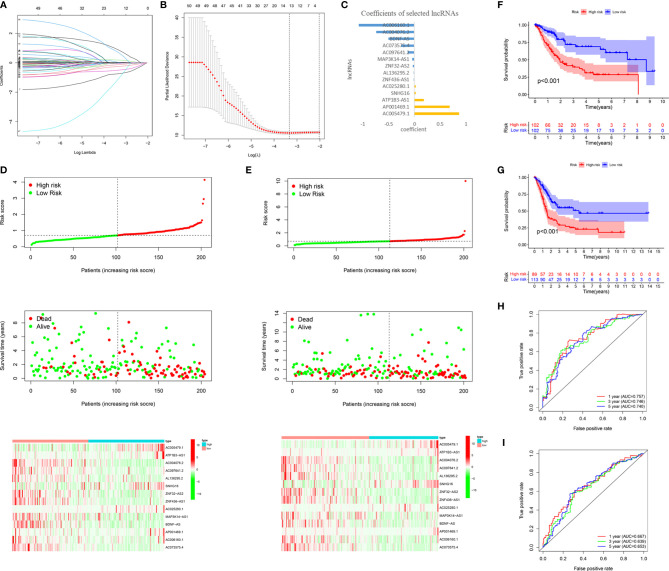
Construction and validation of the m6A-LPS in the TCGA cohort. **(A–C)** LASSO regression analysis was performed, and the minimum criteria **(A, B)** and coefficients **(C)** were calculated. **(D, E)** Distribution of risk scores, OS rates, and OS statuses and the heatmap of the 14 prognostic m6A-related lncRNAs in the TCGA training cohort **(D)** and the TCGA validation cohort **(E)**. **(F, G)** Kaplan-Meier curves of OS rates for bladder cancer patients based on the risk scores in the TCGA training cohort **(F)** and the TCGA validation cohort **(G)**. **(H, I)** Time-dependent ROC curves for the TCGA training cohort **(H)** and the TCGA validation cohort **(I)**.

To accurately predict the clinical prognosis of m6A-related lncRNAs in BC patients, the 50 lncRNAs related to m6A genes were analyzed by LASSO Cox regression. We identified the m6A-LPS, which accounted for the expression levels of the 14 m6A-related lncRNAs and their respective coefficients (**Figures 5A–C**). Then, based on the expression of the 14 lncRNAs and their risk coefficients, we calculated the risk score for each patient in the TCGA training cohort and the validation cohort. We divided patients in the TCGA training and validation cohorts into high-risk and low-risk groups based on the median risk score. **Figures 5D, E** show the risk scores, OS rates, vital statuses, and expression profiles of m6A-LPS components in the TCGA training and validation cohorts. The heatmap indicated that AC005479.1, ATP1B3-AS1, SNHG16, AC025280.1, and AP001469.1 were highly expressed in the high-risk group. In the TCGA training and validation cohorts, there was a significant difference in OS rates between the low-risk and high-risk groups (p < 0.0001, **Figures 5F, G**).

We analyzed the ROC curves for 1-year, 3-year, and 5-year survival by comparing AUC values to evaluate the prognostic accuracy of the 14 risk lncRNAs. We found that the 1-, 3- and 5-year AUC values of the 14-lncRNA signature were 0.757, 0.746, and 0.740, respectively, in the training cohort (**Figure 5H**) and 0.667, 0.639, and 0.653, respectively, in the validation cohort (**Figure 5I**). The AUC values indicated that the 14-lncRNA signature has a good ability to differentiate prognosis in BC patients.

### m6A-LPS Was an Independent Prognostic Factor in Patients With BC

**Figure 6:**
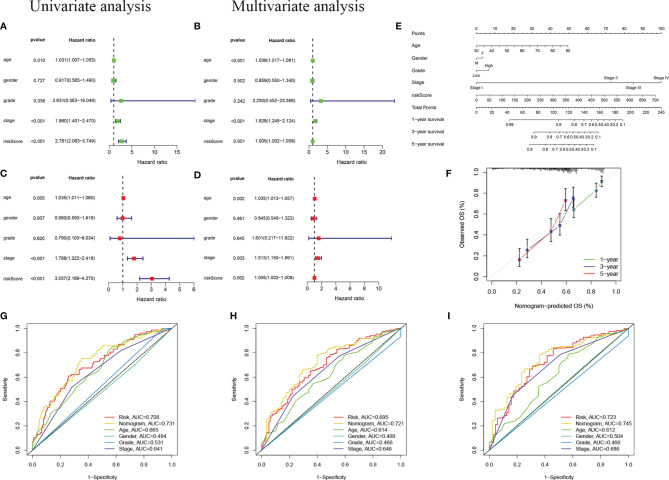
Independent prognostic analysis. **(A, B)** Univariate **(A)** and multivariate **(B)** Cox regression analyses in the TCGA training cohort. **(C, D)** Univariate **(C)** and multivariate **(D)** Cox regression analyses in the TCGA validation cohort. **(E)** Nomogram based on age, gender, grade, stage, and risk score. **(F)** Calibration plot of a nomogram to predict 1-,3- and 5-year OS in the TCGA cohort. The x-axis represents the nomogram-estimated probabilities and the y-axis represents the observed probabilities. **(G–I)** Discriminatory accuracy for predicting OS assessed by receiver operator characteristics (ROC) analysis calculating the time-independent area under the curves (AUCs).1- **(G)**, 3- **(H)**, and 5-year **(I)** in the TCGA cohort.

To confirm whether the risk score based on m6A-LPS can be used as an independent prognostic factor for patients with BC, we performed univariate and multivariate Cox regression analyses on the TCGA training and validation cohorts. Univariate analysis showed that in the TCGA training cohort, age (*P* =0.10), stage (*P <*0.001), and risk score (*P <*0.001) correlated with the OS rate. The multivariate Cox regression analysis of the training cohort showed that age (*P* = 0.005), staging (*P* < 0.001), and risk score (*P* < 0.001) were still closely related to the OS rate (**Figures 6A, B**). Similarly, we conducted the same analysis for the TCGA validation cohort, and the results showed that age, stage, and risk score were independent prognostic factors (**Figures 6C, D**). We found that the risk score based on the m6A-LPS could be used as an independent prognostic factor for the TCGA training and validation cohorts, indicating that the m6A-LPS may have potential value in the process of assessing clinical prognosis.

In addition, we assembled a nomogram based on risk score, age, sex, grade, and stage to predict the prognosis of BC (**Figure 6E**). In our nomogram, the calibration curve has a good ability to predict the prognosis of 1-year, 3-year, and 5-year OS rates (**Figure 6F**). The AUC of the nomogram in the ROC curve is 0.731, 0.721, and 0.745 at 1-, 3-, and 5-year, respectively, which is more accurate than risk score, age, sex, grade, and stage (**Figures 6G–I**). In general, the risk score signature we established can provide the most useful and accurate guidance for predicting the survival of these prognostic indicators.

### Analysis of the Correlation Between the Risk Score and Clinical Characteristics of BC Patients

**Figure 7:**
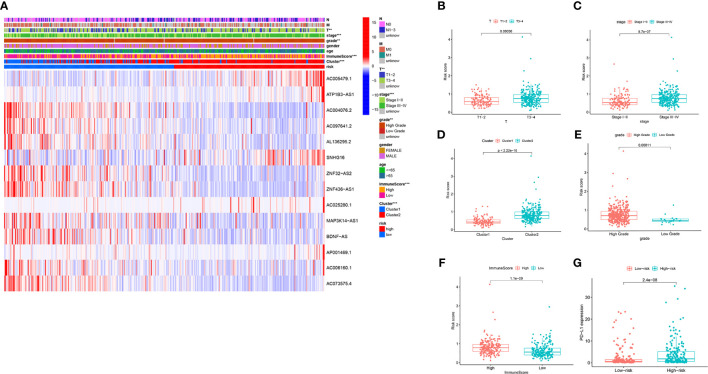
The prognostic risk score was correlated with clinicopathological features and immune score in the TCGA datasets. **(A)** Heatmap and clinicopathologic features for the high- and low-risk groups. **(B–F)** Distribution of risk scores stratified by T stage **(B)**, stage **(C)**, cluster **(D)**, grade **(E)** and, immune score **(F)**. **(G)** PD-L1 expression by risk score group in the TCGA datasets. **P < 0.01; ***P < 0.001.

We further estimated the relationship between the risk score and clinical characteristics in TCGA datasets. The heatmap in **Figure 7A** shows the differences in the expression levels of 14 m6A-related lncRNAs in the TCGA datasets in the low-risk and high-risk groups. There were significant differences in T stage (*P* < 0.01), stage (*P* < 0.001), cluster subtype (*P* < 0.001), grade (*P* < 0.01) and immune score (*P* < 0.001) between the low-risk group and the high-risk group. The risk scores of the T3-4, late-stage, cluster 2, high grade, and high immune score groups were significantly higher than those of the T1-2, early-stage, cluster 1, low grade, and low immune score groups (**Figures 7B–F**). In addition, we found that PD-L1 expression in patients with high-risk scores was significantly higher than that in patients with low-risk scores (*P* < 0.001; **Figure 7G**). These findings revealed that the risk score based on m6A-LPS was significantly associated with subtype, grade, T stage, stage, immune score, and PD-L1 expression in BC patients, also suggesting a potential correlation between the risk score and the TME of BC.

### Immune Cell Enrichment Analysis

**Figure 8:**
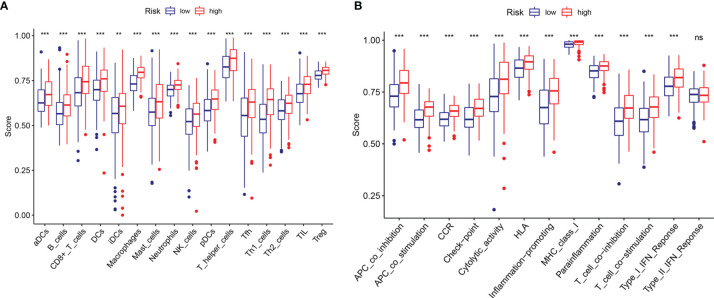
Comparison of the ssGSEA scores between the high- and low-risk groups. The scores for 16 immune cells **(A)** and 13 immune-related functions **(B)** are displayed in boxplots. ns, not significant. **P < 0.01; ***P < 0.001.

To explore the potential correlation between the risk score and the TME of BC, we further studied the correlation between the risk score and immune infiltrating cells and immune functions by ssGSEA in TCGA datasets. We found significant differences in 16 types of immune cells between the high- and low-risk groups (**Figure 8A**): activated dendritic cells (aDCs); macrophages; neutrophils; NK cells; immature dendritic cells (iDCs); dendritic cells (DCs); mast cells; B cells; CD8+ T cells; plasmacytoid dendritic cells (pDCs); T helper cells; T helper 2 (Th2) cells; tumor-infiltrating lymphocytes (TILs); Tregs; T helper 1 (Th1) cells; and follicular T helper (Tfh) cells. In addition, the scores of immune functions, such as cytolytic activity, HLA, cytokine, and cytokine receptor (CCR), T cell coinhibition, T cell costimulation, APC inhibition, APC stimulation, the type I IFN response, and checkpoints, were significantly higher in the high-risk group, implying that these immune functions are more active in patients in the high-risk group (**Figure 8B**). These results indicated that the risk score based on m6A-LPS was closely related to immune infiltrating cells and immune functions in BC, which further suggested that m6A-LPS had a potential influence on the TME of BC.

### Effect of m6A-LPS on Immune Cell Infiltration

**Figure 9:**
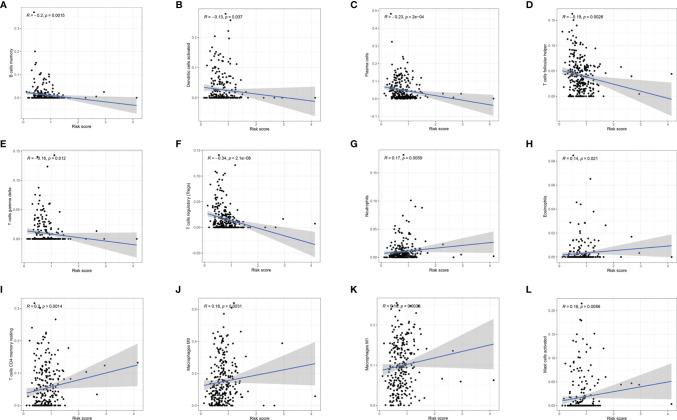
Relationships between the risk score and the infiltration levels of 12 immune cell types. **(A–F)** The infiltration levels of memory B cells **(A)**, aDCs **(B)**, plasma cells **(C)**, Tfh cells **(D)**, gamma delta T cells **(E)**, and Tregs **(F)** were negatively correlated with risk score. **(G–L)** The infiltration levels of neutrophils **(G)**, eosinophils **(H)**, resting memory CD4 T cells **(I)**, M0 macrophages **(J)**, M1 macrophages **(K)**, and activated mast cells **(L)** were positively correlated with the risk score.

To investigate whether m6A-LPS can regulate the TME of BC, we analyzed the correlation between the risk score and the infiltration of 12 types of immune cells in the TCGA datasets. We found that the infiltration levels of memory B cells, activated dendritic cells, plasma cells, follicular helper T cells, and gamma delta Tregs were negatively correlated with the risk score (*P* < 0.05, **Figures 9A–G**). Moreover, the infiltration levels of eosinophils, resting memory CD4 T cells, M0 macrophages, M1 macrophages, and activated mast cells were positively correlated with the risk score (*P* < 0.05, **Figures 9H–L**). Our results indicate that m6A-LPS has pivotal regulatory effects on the TME in BC patients.

### SNHG16 Was Highly Expressed in BC Samples and Affected BC Cell Proliferation, Migration, and Invasion

**Figure 10:**
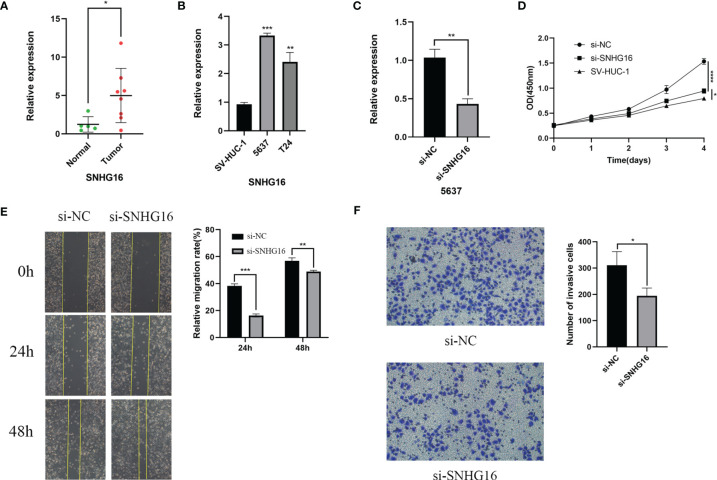
SNHG16 was highly expressed in BC tissues and cell lines and interfered with siRNA resulted in suppressed proliferation, migration, and invasion *in vitro*. **(A)**The SNHG16 expression in BC tissues and paracancerous tissues. **(B)** SNHG16 expression level was assessed in the immortalized human bladder epithelial cell line SV-HUC-1 and the BC cell lines 5637 and T24 by qPCR. **(C)** The efficiency of siRNA knockdown of SNHG16 was assessed in the BC cell line 5637 by qPCR. **(D)** Cell proliferation was determined in SV-HUC-1 cells and 5637 cells transfected with si-SNHG16 or si-NC by CCK-8 assay on days 0, 1, 2, 3, and 4. **(E)** Scratch wound assay: The scratch was imaged after transfection, and cell mobility was measured by determining the extent of wound closure at 24 and 48 h (× 100). **(F)** The invasion of 5637 cells transfected with si-SNHG16 or si-NC was evaluated by transwell assay with Matrigel (× 200). *P<0.05, **P<0.01, ***P<0.001, and ****P<0.0001.

For verification, we detected SNHG16 expression in 13 samples of BC patient tissues, including 8 BC tissues and 5 paracancerous tissues, by RT-qPCR assay. Our results showed that SNHG16 was upregulated in BC tissues compared with normal tissues (*P <*0.05, **Figure 10A**). The result was consistent with those in **Figure 2B**. In addition, we detected SNHG16 expression in the immortalized human bladder epithelial cell line SV-HUC-1 and the BC cell lines 5637 and T24 by qPCR. SNHG16 expression was higher in 5637 and T24 cells than in SV-HUC-1 cells, and its expression level in 5637 cells was higher than that in T24 cells (*P <*0.05, **Figure 10B**). As shown in **Figure 7A**, SNHG16 was highly expressed in the high-risk group, which suggested that SNHG16 promotes the development of BC. Therefore, the role of SNHG16 in BC was further explored. We then selected 5637 cells with higher SNHG16 expression levels for further study. After knocking down SNHG16 by siRNA (**Figure 10C**), our study showed that the proliferation, migration, and invasion ability of BC cell lines with low SNHG16 expression were decreased compared with that in cells with high SNHG16 expression (*P <*0.05, **Figures 10D–F**); however, the proliferation ability of 5637 cells with SNHG16 knockdown was still higher than that of SV-HUC-1 cells (*P <*0.05, **Figure 10D**). Our results showed that SNGH16, which is relatively highly expressed in the high-risk group, is highly expressed in BC tissues and can promote BC cell proliferation, migration, and invasion.

## Discussion

Because of its poor prognosis and high recurrence rate, BC is considered a serious threat to health (31). It is critical to identify specific and sensitive biomarkers to improve the prognosis of patients with BC. Recently, the expression levels and mechanisms of mRNAs and miRNAs have been extensively studied in BC, and many of these factors have been identified as prognostic markers in BC patients (32–36). With further research on BC, the role of lncRNAs has been gradually uncovered and is helpful for comprehensively understanding the characteristics of the occurrence and development of BC (37–39). Many studies have confirmed that m6A methylation may play regulatory roles in the tumorigenesis, development, and progression of BC (40, 41); however, it is not clear how m6A modification plays a role in BC progression in a lncRNA-dependent manner.

We first identified 50 m6A-related lncRNAs with prognostic value in TCGA datasets and then performed consensus clustering on these m6A-related lncRNAs to identify two BC subtypes, namely, cluster 1 and cluster 2. Compared with the cluster 1 subgroup, the prognosis of the cluster 2 subgroup was worse (p = 0.008). Next, we performed GSEA on cluster 1 and cluster 2 to identify the possible major functional pathways. GSEA results showed that hallmarks of malignant tumors (including allograft rejection, apical junction, apoptosis, coagulation, complement, epithelial-mesenchymal transition, late estrogen response, hypoxia, IL2/STAT5 signaling, IL6/JAK/STAT3 signaling, inflammatory response, interferon gamma response, KRAS signaling, mTORC1 signaling, P53 pathway and TNFA signaling *via* NFKB) were significantly enriched in cluster 2, in which the IL6/JAK/STAT3 signaling pathway was shown to induce the expression of PD-1 and/or PD-L1 (29, 30). There are more and more studies on the regulation of PD-L1 in BC. For example, recent studies have shown that WDR5 activates PD-L1 expression through H3K4me3 modification, and OICR-9429 targets WDR5 to inhibit immune escape by blocking PD-L1 (42). PD-L1 has been shown to weaken the antitumor immune response and plays an important role in a variety of tumors (43). Then, we analyzed PD-L1 expression in cluster 1 and cluster 2 and found that PD-L1 was significantly more highly expressed in cluster 2 (p < 0.001), which indicates a correlation between PD-L1 and the IL6/JAK/STAT3 signaling pathway. The analysis revealed an unexpected correlation between PD-L1 expression and m6A-related lncRNAs, suggesting that these specific m6A-related lncRNAs are involved in the development of BC and the expression of PD-L1 in BC through the IL6/JAK/STAT3 signaling pathway. In our study, different subtypes were related to the prognosis and some clinicopathological characteristics of BC and were tightly associated with the expression of PD-L1 and the degree of immune cell infiltration.

Through subtype cluster analysis, we found that the 50 m6A-related lncRNAs we selected were indeed closely related to the prognosis of patients with BC, and this result may provide useful information for predicting the prognosis of BC patients in the clinic. However, more convenient and effective predictors are required; thus, we constructed m6A-LPS. Fourteen prognostic risk factors were obtained by LASSO Cox regression analysis. After correlation calculation, the risk score of each patient was obtained. Among these risk factors, lncRNA SNHG16 showed significantly upregulated expression in hepatocellular carcinoma, lung cancer, colorectal cancer, glioma, and other tumor tissues and cell lines, and the overexpression of SNHG16 often indicates a poor prognosis (44–47). Subsequently, we proved that SNHG16 expression in BC tissues was higher than that in adjacent tissues, and high SNHG16 expression could promote BC cell proliferation and migration, which is consistent with the results of a previous study (48). Our results showed that SNHG16 was highly expressed in the high-risk group, which was consistent with the above analysis.

Then, the patients were subdivided into high- and low-risk groups according to the median value in the training and validation TCGA cohorts. We found that patients in the high-risk group had a poorer prognosis in both cohorts. Based on univariate and multivariate Cox regression analyses, we confirmed that the risk score was an independent prognostic factor for patients with BC. Within the nomogram integrating independent prognostic factors, the risk score made a significant contribution and performed better than other factors in survival prediction. The risk score was also related to the T stage, different cluster subtypes, grade, immune score, and PD-L1 expression level. We also observed that cluster 2 had a higher risk score and that PD-L1 expression was higher in the high-risk group, which was consistent with the findings of previous studies. Moreover, the immune score of the high-risk group was higher than that of the low-risk group. We further explored the relationship between the risk score and immune cells and immune functions by ssGSEA. The results showed that the expression levels of 16 types of immune cells in the high-risk group were significantly higher than those in the low-risk group. Rosenberg et al. (49) found that the expression of PD-L1 in BC was more closely associated with immune infiltrating cells than with tumor cells. In addition, some studies have shown that responses to ICIs are often associated with high tumor mutation loads, resulting in the production of a large number of mutated neoantigens that support extensive immune cell infiltration and render tumors sensitive to ICIs (50, 51). The results of the above analysis may explain the correlation between the high expression of PD-L1 and the high immune cell infiltration in the high-risk group in BC and indicate that ICIs are more effective for patients in the high-risk group.

Immune cell infiltration in the TME may affect the survival, metastasis, and therapeutic resistance of tumor patients (52–54). Previous studies have shown that neutrophils in tumor immune cell infiltration are associated with a poor prognosis (50, 55), while BC patients with high levels of Treg infiltration have a better prognosis (56). In this study, the risk score was negatively correlated with activated dendritic cells, plasma cells, memory B cells, Tfh cells, gamma delta T cells, and Tregs and positively correlated with neutrophils, eosinophils, resting memory CD4 T cells, M0 macrophages, M1 macrophages, and activated mast cells. Taken together, these results suggest that the 14 risk m6A-related lncRNAs in BC were associated with the infiltration of these immune cell subtypes. Our results were consistent with previous results.

However, our results need to be further verified before they can be used to assist the clinical treatment of patients with BC, such as determining whether it is appropriate to apply PD-L1 blockade therapy and to finally achieve the purpose of predicting and improving the prognosis of patients with BC. Moreover, the different immune characteristics of the subtypes established by the m6A-LPS classification system indicate that patients from different subgroups may have different responses to immunotherapy. More studies are needed to confirm the accuracy of the prediction system and whether personalized treatment for these subtypes can improve patient prognosis.

## Conclusions

In summary, this study methodically assessed the prognostic value of m6A-related lncRNAs in BC from TCGA datasets and studied the correlation between these lncRNAs and PD-L1 expression and the TME. m6A-related lncRNAs might be involved in the regulation of PD-L1 expression and the TME of BC in synergy with the IL6/JAK/STAT3 signaling pathway. The risk score based on the m6A-LPS was shown to be an independent prognostic indicator and could effectively predict the prognosis of patients with BC. Therefore, identifying m6A-related lncRNAs related to the signaling pathway affecting the TME and further studying their regulatory mechanism may provide a promising target for improving the responsiveness of BC to immunotherapy.

## Data Availability Statement

The original contributions presented in the study are included in the article/**Supplementary Material**. Further inquiries can be directed to the corresponding authors.

## Ethics Statement

The studies involving human participants were reviewed and approved by the Medical Ethics Committee of Renmin Hospital of Wuhan University. The patients/participants provided their written informed consent to participate in this study.

## Author Contributions

KC designed this study. KC, SZ, WY, and YX performed the data analysis, plotted the figures, and wrote the manuscript. JX revised the content. JG and FC were responsible for confirming the authenticity of the data. All the authors read and approved the final manuscript.

## Funding

The current study was funded by the Algorithm and Application of Intelligent Medical Service based on health-related big data (Grant No. 2019AEA170).

## Conflict of Interest

The authors declare that the research was conducted in the absence of any commercial or financial relationships that could be construed as a potential conflict of interest.

## Publisher’s Note

All claims expressed in this article are solely those of the authors and do not necessarily represent those of their affiliated organizations, or those of the publisher, the editors and the reviewers. Any product that may be evaluated in this article, or claim that may be made by its manufacturer, is not guaranteed or endorsed by the publisher.
