# Corrigendum: Comprehensive Analysis of N6-Methylandenosine-Related Long Non-Coding RNAs Signature in Prognosis and Tumor Microenvironment of Bladder Cancer

**DOI:** 10.3389/fonc.2022.908832

**Published:** 2022-05-06

**Authors:** Kang Chen, Shaoming Zhu, Weimin Yu, Yuqi Xia, Ji Xing, Jie Geng, Fan Cheng

**Affiliations:** ^1^ Department of Urology, Renmin Hospital of Wuhan University, Wuhan, China; ^2^ Department of Urology, Suizhou Hospital, Hubei University of Medicine, Suizhou, China

**Keywords:** bladder cancer, N6-methyladenosine, prognostic signature, immune infiltration, long non-coding RNA

In the original article, there was a mistake in the legend for **Figure 7F** as published. When we changed the size of the pictures according to the requirements of the reviewers, due to our negligence, we repeated the **Figure 7G** layer in the PS software, so that the original **Figure 7F** is overwritten by repeated **Figure 7G**, resulting in the **Figure 7F** not appearing correctly. The corrected **Figure 7** legend appears below

**Figure 7:**
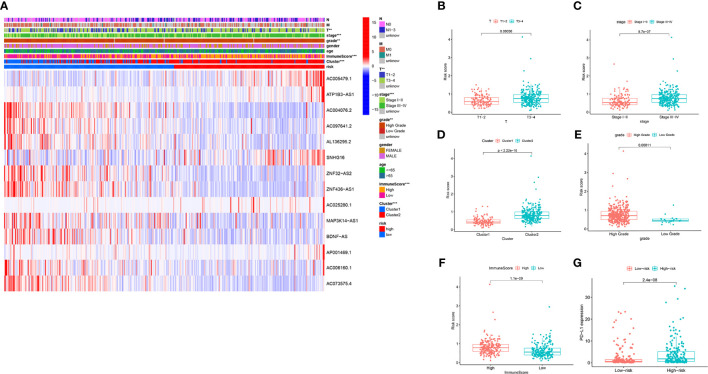
The prognostic risk score was correlated with clinicopathological features and immune score in the TCGA datasets. **(A)** Heatmap and clinicopathologic features for the high- and low-risk groups. **(B–F)** Distribution of risk scores stratified by T stage **(B)**, stage **(C)**, cluster **(D)**, grade **(E)** and, immune score **(F)**. **(G)** PD-L1 expression by risk score group in the TCGA datasets. **P < 0.01; ***P < 0.001.

The authors apologize for this error and state that this does not change the scientific conclusions of the article in any way. The original article has been updated.

## Publisher’s Note

All claims expressed in this article are solely those of the authors and do not necessarily represent those of their affiliated organizations, or those of the publisher, the editors and the reviewers. Any product that may be evaluated in this article, or claim that may be made by its manufacturer, is not guaranteed or endorsed by the publisher.

