# Supplementary material for: Comprehensive Analysis of N6-Methylandenosine-Related Long Non-Coding RNAs Signature in Prognosis and Tumor Microenvironment of Bladder Cancer
Source: Front Oncol. 2022 Jan 24;12:774307. doi: 10.3389/fonc.2022.774307 (PMC8818872; doi:10.3389/fonc.2022.774307)
Supplement: Supplementary file 1 [file Table_1.docx]

Supplemental information

Table S1. m6A-related lncRNAs with significant prognostic value in bladder cancer identified by Cox regression analysis.

| gene | HR | HR.95L | HR.95H | pvalue |
| --- | --- | --- | --- | --- |
| AC005479.1 | 2.407019 | 1.356165 | 4.272151 | 0.002693 |
| TMEM147-AS1 | 0.866778 | 0.78906 | 0.952151 | 0.002855 |
| ATP1B3-AS1 | 1.207264 | 1.090085 | 1.33704 | 0.000299 |
| AC087286.2 | 1.45984 | 1.102158 | 1.933601 | 0.008333 |
| AC104532.2 | 0.61745 | 0.458863 | 0.830847 | 0.001455 |
| SPAG5-AS1 | 0.39168 | 0.198595 | 0.772493 | 0.006833 |
| AC004076.2 | 0.265562 | 0.109636 | 0.643249 | 0.003309 |
| AL161452.1 | 0.463899 | 0.259037 | 0.830778 | 0.009779 |
| SNHG20 | 0.823183 | 0.721895 | 0.938682 | 0.003678 |
| U47924.1 | 0.586282 | 0.390935 | 0.879242 | 0.009812 |
| AC068768.1 | 0.297793 | 0.119351 | 0.743026 | 0.009413 |
| AC116914.2 | 0.703808 | 0.586719 | 0.844262 | 0.000155 |
| PTOV1-AS2 | 0.884188 | 0.825417 | 0.947144 | 0.000453 |
| AC097641.2 | 0.666358 | 0.490307 | 0.905624 | 0.009507 |
| AL136295.2 | 0.483638 | 0.324345 | 0.721163 | 0.000366 |
| AC132872.3 | 0.792858 | 0.672363 | 0.934948 | 0.005785 |
| AC005306.1 | 0.432242 | 0.242316 | 0.771029 | 0.004503 |
| AC104564.3 | 0.595067 | 0.437124 | 0.810077 | 0.000973 |
| SNHG16 | 1.087945 | 1.022312 | 1.157791 | 0.00793 |
| AC012568.1 | 1.245505 | 1.073481 | 1.445094 | 0.003792 |
| ZNF32-AS2 | 0.695796 | 0.539914 | 0.896683 | 0.005069 |
| ZNF436-AS1 | 0.868946 | 0.782873 | 0.964483 | 0.008304 |
| KCNQ1OT1 | 2.155119 | 1.384412 | 3.35488 | 0.000673 |
| AL022322.1 | 0.816873 | 0.721988 | 0.924228 | 0.001324 |
| RAP2C-AS1 | 3.023214 | 1.327917 | 6.882829 | 0.008398 |
| AC007686.3 | 0.168875 | 0.045886 | 0.621513 | 0.007465 |
| EHMT2-AS1 | 0.278015 | 0.137558 | 0.561888 | 0.000363 |
| AC012615.6 | 0.561941 | 0.379539 | 0.832003 | 0.003996 |
| LINC02604 | 0.9036 | 0.848175 | 0.962646 | 0.001697 |
| AC097359.2 | 1.732258 | 1.151868 | 2.605088 | 0.008313 |
| Z84485.1 | 0.471937 | 0.28503 | 0.781409 | 0.003515 |
| ZNF32-AS1 | 0.656139 | 0.483146 | 0.891072 | 0.006964 |
| AL138756.1 | 0.7264 | 0.574601 | 0.918301 | 0.007528 |
| AC025280.1 | 1.974099 | 1.284462 | 3.034008 | 0.001925 |
| AC073534.2 | 0.573345 | 0.412422 | 0.797058 | 0.000935 |
| MAP3K14-AS1 | 0.679795 | 0.517732 | 0.892587 | 0.005473 |
| AC022150.2 | 0.85558 | 0.772347 | 0.947782 | 0.002817 |
| AL022328.2 | 0.828023 | 0.73071 | 0.938297 | 0.003092 |
| AC074117.1 | 0.829424 | 0.72927 | 0.943331 | 0.004393 |
| AC020911.1 | 0.198618 | 0.066933 | 0.589375 | 0.003584 |
| AL138921.1 | 0.180975 | 0.060069 | 0.545239 | 0.002383 |
| BDNF-AS | 0.390199 | 0.211924 | 0.718444 | 0.002514 |
| AC008764.8 | 0.363002 | 0.169774 | 0.77615 | 0.008961 |
| THAP9-AS1 | 0.938861 | 0.896009 | 0.983762 | 0.008126 |
| AC004148.1 | 0.831239 | 0.74787 | 0.923902 | 0.000609 |
| AP001469.1 | 1.947167 | 1.203181 | 3.151197 | 0.006667 |
| AC006160.1 | 0.185773 | 0.054274 | 0.635882 | 0.007338 |
| LINC00115 | 0.621664 | 0.437198 | 0.88396 | 0.008127 |
| AC010201.2 | 0.574756 | 0.380466 | 0.868264 | 0.008512 |
| AC073575.4 | 0.554452 | 0.374251 | 0.82142 | 0.003272 |
